# Acoustic Wave Propagation Behaviors and Energy Loss Mechanisms in Agar Gels with Small Particles

**DOI:** 10.3390/polym17162226

**Published:** 2025-08-15

**Authors:** Yuqi Jin, Teng Yang, Yunlong Qi

**Affiliations:** 1 Department of Mechanical Engineering, University of North Texas, Denton, TX 76207, USA; 2 Department of Physics, University of North Texas, Denton, TX 76203, USA; 3 Department of Materials Science and Engineering, University of North Texas, Denton, TX 76207, USA; tengyang@my.unt.edu; 4 Department of Electrical Engineering, University of North Texas, Denton, TX 76207, USA; yunlongqi@my.unt.edu

**Keywords:** hydrogel, tissue phantom, attenuation, ultrasound, acoustic property modification, pulse distortion

## Abstract

Soft organic gels are commonly used as tissue phantoms for experiments. In the mimic ultrasound imaging field, researchers are developing approaches to modify the acoustic properties of the gels. Introducing oil liquids and hard solid particles are two common methods to tune acoustic and mechanical properties of the soft gels. In this work, the acoustic wave energy loss mechanisms were studied in detail on Agar gel with both micro-Graphite and nano-Alumina particles. Via experimental measurements, the results show that the effective acoustic energy loss is comparable in these two recipes. However, temporal pulse elongation and scattering behaviors were distinguishable. To understand the sound attenuation mechanism in detail, numerical simulations in controlled conditions were conducted, from wavelengths longer than the particle diameter to wavelengths shorter than particles, and we compared perfect bonding and insufficient bonding between the hard particles surrounding gels. Comparing the experimental observations and numerical simulation results, the Agar gel with nano-Alumina presents stronger dispersion-induced energy loss than the Agar gel with micro-Graphite. On the contrary, the Agar gel with micro-Graphite shows more significant scattering-induced destructive interferences than the Agar gel with nano-Alumina.

## 1. Introduction

In the human body, soft tissues such as fat, muscle, and ligaments play a crucial role in connecting, supporting, and protecting organs [1]. Due to challenges in using natural tissue, including high cost, biological inconsistency, and preservation difficulties, tissue phantoms have become vital tools for biomedical research [2]. These synthetic models are designed to replicate the mechanical, acoustic, and physiological properties of human tissues, enabling the development and calibration of diagnostic tools like ultrasound detection mapping [3,4] and ultrasound imaging [5,6]. Particularly in clinical settings involving a high body mass index or fluid accumulation, the strong dispersion and dissipation of acoustic waves in soft tissues pose significant challenges for diagnostic accuracy [7,8]. To enhance the biomimetic capabilities of tissue phantoms, researchers have increasingly explored the use of additives, liquids and/or particles to fine-tune their acoustic and mechanical properties. Base phantom materials such as gelatin [9,10], agarose (Agar) [11,12], and silicone rubber [13,14] are chosen due to their mechanical resemblance to soft biological tissues and ease of fabrication. Gelatin and Agar gels are historically the most widely used because they closely mimic soft tissue elasticity and acoustic impedance, are cost-effective, and can be easily molded into desired shapes and sizes. Agarose, a natural polysaccharide derived from seaweed, offers unique benefits such as superior biocompatibility, thermo-reversible gelation, and consistent physicochemical characteristics, making it particularly suitable for tissue engineering and biomedical imaging applications. Silicone rubber-based gels, by contrast, are primarily employed in phantoms designed to emulate muscle tissues and breast tissues due to their flexible mechanical profile and stable acoustic response.

Beyond these base materials, scientists often introduce secondary ingredients to alter the acoustic behavior of the phantom to better match various types of human tissue. For instance, the addition of evaporated milk into gelatin has been shown to modify its acoustic attenuation and scattering characteristics due to its protein and fat content, making the phantom more representative of fatty tissues [15]. Similarly, mineral oil is incorporated into silicone rubber to modulate the speed of sound and attenuation coefficient, emulating tissues with higher lipid content or lower density [16]. The concentration of these additives can be carefully controlled to simulate a range of tissue environments from lean muscle to adipose-dense regions. Graphite or Al_2_O_3_ particles have also often been added to the tissue phantom for stronger acoustic absorption or attenuation. However, different from liquid ingredients, adding micro-particles does not provide repeatability on mechanical properties or acoustic properties, due to the difference in the size and distribution of the particles and the operation acoustic wavelength. Nevertheless, this adaptability allows researchers to build customized phantoms tailored to specific diagnostic challenges, such as acoustic imaging through fat, fluid-rich, or fibrosis anatomical regions that show significantly different acoustic properties and wave propagation behaviors.

Among all the acoustic properties, speed of sound, impedance, attenuation, back-scattering coefficient, and nonlinearity parameters have been extensively studied [17,18]. Acoustic attenuation, the reduction in the amplitude of sound waves as they propagate through a medium, is often considered a critical determinant of ultrasound imaging quality [19]. However, this parameter alone does not fully explain the degradation in image clarity [20,21], especially in complex biological environments. Although attenuation primarily reduces the signal-to-noise ratio, the dispersion of acoustic waves is also caused by the elastic and viscoelastic properties of the tissue [22] that lead to temporal pulse broadening [23,24]. This dispersion affects [25] the temporal resolution of ultrasound systems and, consequently, the sharpness and diagnostic utility of the resulting images, which was discussed in detail in the previous study [21]. Regardless of its significant implications, dispersion has received far less attention in the literature compared to parameters like the speed of sound, acoustic impedance, and the backscatter coefficient, especially with complex gel recipes. In this context, our study aims to fill this gap by closely discovering the detailed energy loss mechanisms across two commonly used soft tissue Agar phantoms with some small particles including micro-Graphite [26,27] or nano-Al_2_O_3_ [28,29].

In the literature, most ultrasonic characterization studies have estimated an increase in attenuation coefficients of tissue phantoms by introducing micro-Graphite [26,27] or nano-Al_2_O_3_ particles [28,29], often using bistatic immersion setups or direct monostatic setups. However, the results indicate the potential existence of conditions including weaker bonding between tissue phantoms and a non-negligible scattering effect due to the close size ratio between the wavelength and the size of the particles. On the contrary, with strong bonding between tissue phantoms, a longer wavelength acoustic wave should experience less attenuation with the introduction of these microparticles due to the increased effective density and bulk modulus according to the effective medium approximation [23]. In particular, when the scattering and dispersion effects are significant, the distortion of the acoustic pulse envelopes makes the conventional attenuation coefficient estimation inaccurate or even invalid. To address the potential experimental results mentioned, we employed numerical simulations and experimental validations to compare the performance of the conventional monostatic acoustic energy loss measurement with a fixed particle size under different fundamental frequency acoustic pulses in this study.

In a previous report, not considering energy absorption, the significance of dispersion-induced out-of-phase interference was discussed in detail [30] in tissue phantoms with 10 wt.% liquid additives, which strongly increased the overall attenuation effect. Unlike solid small particles, these liquid additives in hydrogel are either dissolved or broken down into liquid cavities, which are usually evenly distributed or appear discontinuous in a small scale. On the contrary, most solid particles have a certain minimum breakdown size and certain bonding conditions, which present case-by-case particle-dependent and frequency-dependent effects on the contribution of acoustic scattering and acoustic dispersion, both simultaneously leading to acoustic energy loss [31,32]. Hence, in this study, multiple particle-dependent and frequency-dependent numerical simulations under controlled conditions were conducted, from wavelengths longer than the particle diameter to wavelengths shorter than particles, and we compared the perfect bonding condition and insufficient bonding condition between the particles and gels. Comparing the experimental observations and numerical simulation results, the Agar gel with 10 wt.% nano-Alumina offers stronger dispersion-induced energy loss than the Agar gel with 10 wt.% micro-Graphite. On the contrary, the Agar gel with micro-Graphite provides more significant scattering-induced destructive interferences than the Agar gel with nano-Alumina.

## 2. Methods

### 2.1. Experimental Setup

**Figure 1:**
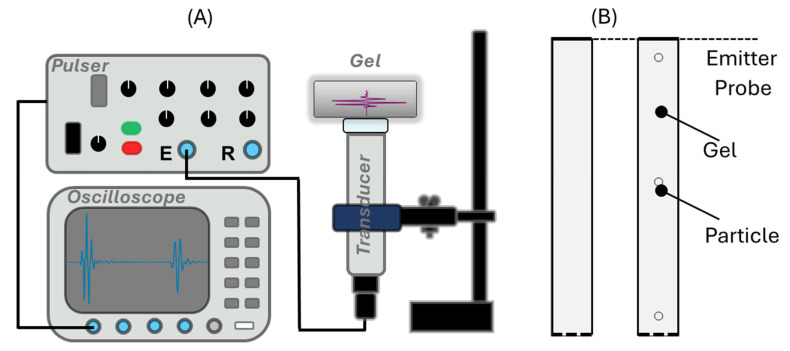
(**A**) Monostatic experimental setup for measuring attenuation in Agar gels. (**B**) Model setup for numerical simulations.

For measurement, an JSR 500 pulse/receiver (JSR Corporation, Tokyo, Japan) and Tek 3024b 2.5 GHz oscilloscope (Tektronix, Beaverton, OR, USA) were used to generate and acquire data in an averaging (512) mode in the temporal domain. Olympus 1 MHz 1” V302 transducers (Olympus, Tokyo, Japan) were used in a monostatic setup (Figure 1A). The dispersion and attenuation in the monostatic (pulse echo) arrangement required gain on the pulse echo, resulting in significant signal distortion. Pulses were measured around 22.1 °C (room temperature) in an environment-controlled lab. The sound propagation speed in the samples using standard time-of-flight techniques was calculated from the ratio of specimen thickness to sound wave transit time. The accuracy of the setup was verified for DI water.

Agar is a polysaccharide vegetarian substitute for gelatin extracted from red algae, such as Gracilaria and Gelidium. Agar powder (ECO TASTE, Nanjing, China), 100% nature seaweed, was used to make agar gels with 10 wt.% concentrations. For 10 wt.% Agar gels, 10 g of Agar powder was dissolved in 100 g de-ionized and degassed water, and the mixture was heated up to the boiling point and maintained for 2 min with constant stirring [12]. For the gels with particles, 10 g of Agar powder was dissolved in 100 g, de-ionized and degassed water/particle mixtures, and the mixture was heated up to the boiling point and maintained for 2 min with constant stirring. The micro-graphite and nano-Alumina were purchased from Sigma-Aldrich (St. Louis, MO, USA) and MSE supplies (Tucson, AZ, USA), respectively. The mixture was then cooled down at room temperature for 24 h. We use the same six-well plates (NEST, Wuxi, China) to mold the Agar gel phantom. The 10 wt.% Agar gel follows the same procedure. Just use 10 g of Agar powder and 100 g of water.

### 2.2. Numerical Simulation

In this study, all the numerical simulations were performed by finite element analysis (FEA)-based numerical simulation software: COMSOL Multiphysics 6.0 (COMSOL, Burlington, MA, USA). The pulse waveform in the simulation was expressed as $x\left(t\right)=\sin\left({2\pi f}_{0}t\right)e^{-f_{0}{\left(t-\delta T_{0}\right)}^{2}}$, where $f_{0}$, $T_{0}$ and δ are the fundamental frequency, period, and delay of the pulse, which have been described in previous reports [33,34,35]. All the temporal domain numerical simulation models were studied in a time range from 0 to 100$T_{0}$ with 0.2$T_{0}$ intervals. The numerical simulation cases can be grouped into 2 cases, verification and study (Figure 1B). Verification includes a comparison between the blank gel model and the gel model with 3 particles in the direction of wave propagation. This is worth noting for observing the potential uncertainty induced by the introduction of 3 particles and the corresponding mesh difference. In the verification step, the 3 particles were modeled as gel material, which had identical properties as the surrounding region, known as impedance transparency [36,37]. The acoustic emission source and boundary detection probe were located on the same side at the top of the gel region. The outer boundary of the sample in the monostatic simulation was set up to mimic the hard boundary, creating a reflection boundary condition implemented to eliminate energy radiation, which was comparable to the practical sample/air impedance mismatch condition in the experiments. The Agar tissue phantom sample in the numerical simulation was set with speed of sound, density, and uniform attenuation coefficient values as 1450 m/s, 1198 kg m^−3^, and 5 dB/cm, respectively.

## 3. Results

**Figure 2:**
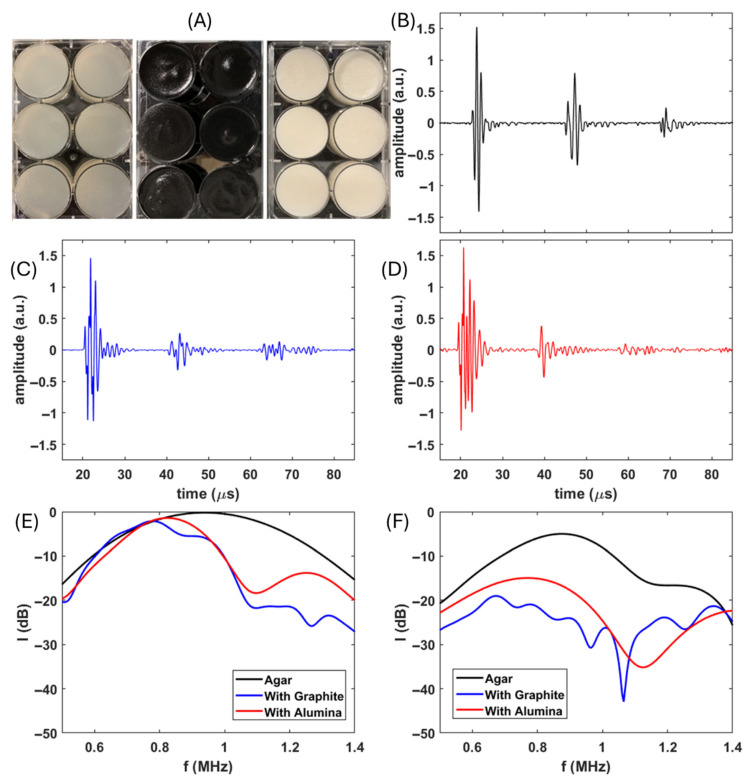
(**A**) Samples for acoustic measurements. From left to right, pure Agar gels, Agar gels with 10 wt.% micro-Graphite, and Agar gels with 10 wt.% nano-Alumina. (**B**) Example temporal waveform from pure Agar gels. (**C**) Example temporal waveform from pure Agar gels with 10 wt.% micro-Graphite. (**D**) Example temporal waveform from pure Agar gels with 10 wt.% nano-Alumina. (**B**) shares an identical time axis with (**C**,**D**). (**E**) Frequency spectrums of the 1st pulse envelopes of each sample. (**F**) Frequency spectrums of the 2nd pulse envelopes of each sample.

To experimentally obtain the attenuation performance of the Agar gels and the gels with particles, fresh samples were made including Agar gel disk samples (Figure 2A, left), Agar gel disks with 10 wt.% Graphite micro-particle samples (Figure 2A, middle), and Agar gel disks with 10 wt.% Al_2_O_3_ nano-particle samples (Figure 2A, right). By using the experimental setup shown in Figure 1B, the multi-roundtrip time-of-flight data were captured and are presented in Figure 2B (Agar), Figure 2C (Agar with Graphite), and Figure 2D (agar with Alumina). It is clear that the pulse waveform in pure Agar gels shows a clean and short pulse in the first and second roundtrip and does not present significant dispersion effects and scattering effects. After the second roundtrip, the waveform presented some scattering-like effects, which were potentially due to the internal reflection around the side wall [21]. In Figure 2C, the roundtrip pulses from the Agar gels with 10 wt.% Graphite micro-particles presented clear scattering effect- and dispersion effect-induced pulse elongation from the first roundtrip pulse. On the contrary, in Figure 2D, the roundtrip pulses from the Agar gels with 10 wt.% Alumina nano-particles only presented clear dispersion effect-induced pulse elongation from the first roundtrip pulse with the absence of a significant scattering waveform.

Figure 2E presents the frequency spectrums of the first roundtrip pulses from the above time domain waveform via Fourier transformation. As mentioned, the short and clean pulse from pure Agar gel provides a Gaussian-like frequency spectrum that follows the typical frequency response of the involved ultrasound transducer. On the contrary, the other samples with particles did not show the complete frequency spectrum from the transducer due to some destructive interferences from scattering or dispersion effects, which aligns with the observation in the time domain waveform. Figure 2F presents the frequency spectrums of the second roundtrip pulses from the above time domain waveform via Fourier transformation for all three selected samples. The second roundtrip pulse from pure Agar gel also presented minor interference, which induced an imperfect Gaussian-like frequency spectrum. Although both time domain data and frequency domain data presented similar attenuation performances among nano-Alumina Agar gel and micro-Graphite Agar gel, compared with the pulse waveforms from Agar with micro-Graphite and Agar with nano-Alumina, it is more obvious, with respect to the first roundtrip pulses in Figure 2E, that the smooth frequency spectrum from the nano-Alumina Agar sample indicates that the dispersion effects contributed the most to acoustic energy loss compared to the scattering effect.

On the contrary, the non-smooth frequency spectrum from the micro-Graphite Agar sample suggests that the scattering effects dominated the contribution to acoustic energy loss compared to the dispersion effect. It is well known that nano-particles in polymers usually provide better bonding compared to micro-particles. Although Alumina has much more mismatching mechanical properties with Agar gel compared with Graphite, the scattering effects are more significant in micro-Graphite Agar gel due to the worse Graphite–Agar bonding. The smaller size and better bonding of nano-Alumina in the Agar gel renders the acoustic wave propagation behavior closer to that of the homogenized medium, which majorly provides dispersion-induced frequency-dependent speed of sound, resulting in the elongation of the pulse and corresponding energy loss.

**Figure 3:**
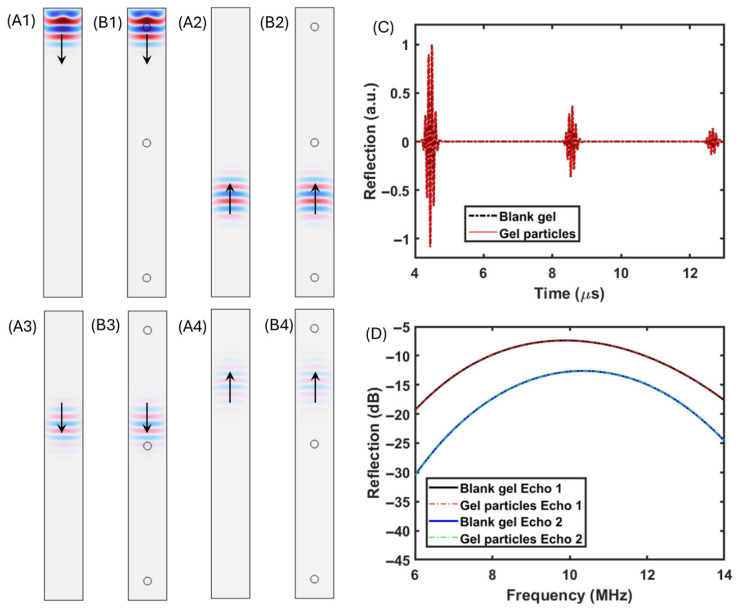
(**A1**–**A4**) Numerical simulation model of pure gel and results in terms of time-dependent acoustic wave propagation behavior. (**B1**–**B4**) Numerical simulation model for verification of gel with 3 gel particles and results in terms of time-dependent acoustic wave propagation behavior. (**C**) Monostatic collected numerical simulation results in time domain. (**D**) Monostatic collected numerical simulation results in frequency domain via Fourier transformation time domain results.

To further understand the difference between the above-mentioned wave propagation behaviors, the following numerical simulations were conducted to offer wave propagation studies under controlled conditions. As the first step of the numerical simulation study, a comparison between a pure gel block and a gel block containing particles was conducted (Figure 3). To ensure that the geometrical difference and induced mesh distribution difference did not create uncertainty in the wave propagation behaviors, the first step of study used gel–particle material to eliminate the mechanical property difference between the particles and the surrounding material. Hence, physically, the models in Figure 3(A1,B1) are identical, with identical material in the gel region and particle regions in Figure 3(B1).

From Figure 3(A1,B1) to Figure 3(A4,B4), the acoustic pressure distribution maps depict the wave propagation behaviors including the initial pulse emission (Figure 3(A1,B1)), the reflected pulse traveling forward to complete the first roundtrip (Figure 3(A2,B2)), the reflected pulse completing the first roundtrip and traveling forward for the second roundtrip (Figure 3(A3,B3)), and the pulse emitted to complete the second roundtrip. From the acoustic pressure distribution, there was no noticeable difference between the compared cases, with only geometrical differences. With the probe overlapping the emission source location (the monostatic setup), the time domain pulse waveforms were collected and are presented in Figure 3C, and they were also transformed into the frequency domain (Figure 3D). It is clear that the numerical simulation models were properly setup without noticeable uncertainty in both the first roundtrip reflection and second roundtrip reflection induced by the introduction of small particles along the wave propagation direction.

**Figure 4:**
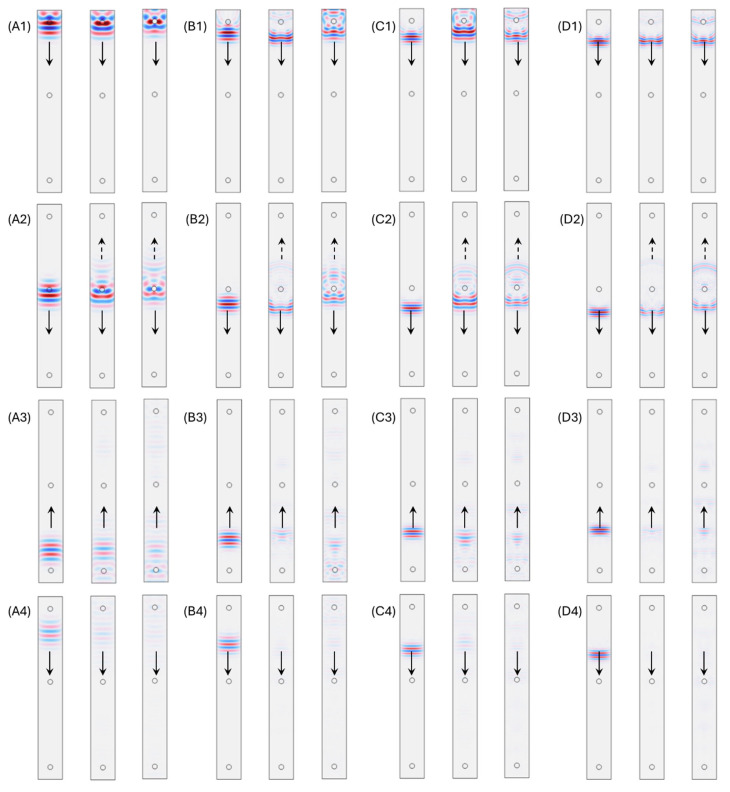
(**A1**–**A4**) Time-dependent acoustic pressure distribution maps under the condition of a wavelength to particle diameter ratio of 1.8. In each subfigure, the left, middle, and right columns refer to the gel–particle reference, perfect bonding hard particles, and insufficient bonding hard particles, respectively. (**B1**–**B4**) Time-dependent acoustic pressure distribution maps under the condition of a wavelength to particle diameter ratio of 1.3. (**C1**–**C4**) Time-dependent acoustic pressure distribution maps under the condition of a wavelength to particle diameter ratio of 0.8. (**D1**–**D4**) Time-dependent acoustic pressure distribution maps under the condition of a wavelength to particle diameter ratio of 0.3.

After the verification of the model setup, the following numerical simulation studies used particles with mechanical properties (the speed of sound and density) that differed to those of the surrounding material (Agar gel). For comparison, in Figure 4, each subfigure shows three cases including gel particles for reference (Case 1), hard particles with perfect bonding (Case 2), and hard particles with insufficient bonding (Case 3). Furthermore, four different operation frequency ranges were selected at 9 MHz, 13 MHz, 17 MHz and 21 MHz, which meant that the ratios (λ/d) between the particle diameter (d) and fundamental frequency wavelength (λ) were about 1.8, 1.3, 0.8, and 0.3.

In Figure 4, each column of the subfigures presents four moments in time domain wave propagation behavior including emission, 0.25 roundtrip, around 1.2 roundtrip, and around 2.2 roundtrip. After the emission of the pulses (Figure 4(A1,B1,C1,D1)), the existence of hard particles in front of the emission source clearly changed the acoustic pressure distribution of the propagating pulses. With a longer operating wavelength (Figure 4(A2,A3,A4)), the good bonding hard particle (Case 2) elongated major pulses and created a minor scattering wave. On the contrary, the insufficient bonding hard particles (Case 3) created stronger phase mismatching in the elongated major pulse with respect to Case 2 (Figure 4(A2,A3)). Compared with Case 2 and Case 3 in Figure 4(A4), with identical hard particles, insufficient bonding between hard particles and the surrounding gel caused slightly more acoustic energy loss.

When the operational wavelength is comparable to the hard particle size (Figure 4(B1–B4,C1–C4)), the major pulse elongation is reduced with stronger scattering and backscattering behavior occurrences (Figure 4(B2,B3,C2,C3)), which leads to more significant acoustic energy loss. With the operational wavelength shorter than the internal particle size (Figure 4(D1–D4)), after the major pulse was propagated through the hard particles, an additional reflection occurred instead of the generation of backscattering. There was no clear pulse elongation observed on the pulse. The difference between perfect bonding and insufficient bonding between the hard particles and surrounding gel offered different amounts of acoustic impedance mismatching, which induced different amplitudes of internal reflections from the hard particles.

**Figure 5:**
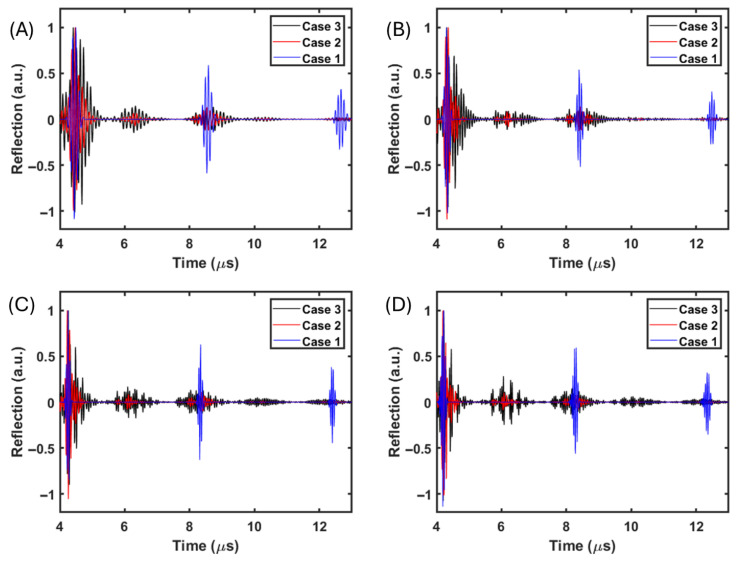
(**A**) Time domain monostatic waveform under the condition of a wavelength to particle diameter ratio of 1.8. In each subfigure, the left, middle, and right columns refer to the gel–particle reference (Case 1), perfect bonding hard particles (Case 2), and insufficient bonding hard particles (Case 3), respectively. (**B**) Time domain monostatic waveform under the condition of a wavelength to particle diameter ratio of 1.3. (**C**) Time domain monostatic waveform under the condition of a wavelength to particle diameter ratio of 0.8. (**D**) Time domain monostatic waveform in the condition of a wavelength to particle diameter ratio of 0.3.

Figure 5 presents the collected time domain signals via the monostatic setup from the above-mentioned numerical simulation studies, where Figure 5A–D correspond to the columns in Figure 4. Comparing Figure 5A–D, the increased operating center frequency induced the shortening of the pulse (Case 1). Around 4 μs and 8 μs, the first roundtrip and second roundtrip pulses can be observed. When the operational wavelength is longer than the particle size (Figure 5A), clear pulse elongation can be observed on both the first and second roundtrip pulse (Case 2 and Case 3). Between the first roundtrip and second roundtrip pulses, a backscattering envelop could be observed at around 6 μs, which was more significant with insufficient bonding than in the perfect bonding case. In Figure 5B and 5C, clear pulse elongation can be observed on both first and second roundtrip pulses (Case 2). Regarding insufficient bonding (Case 3), instead of pulse elongation, it is clear that the main first roundtrip pulse was separated into at least three envelopes. Correspondingly, the backscattering (Figure 5B) and internal reflection (Figure 5C) envelopes in Case 3 (around 6 μs) also experienced clear separation. With a shorter operational wavelength (Figure 5D), similar to Case 3, the first roundtrip pulse in Case 2 was also separated into at least three envelopes at comparable temporal locations with respect to Case 3.

**Figure 6:**
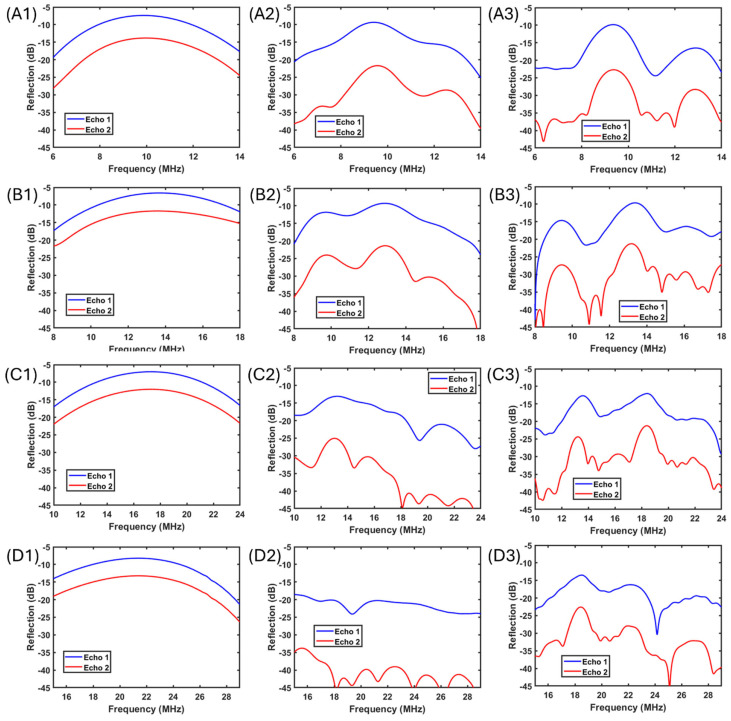
(**A1**–**A3**) Frequency domain monostatic waveform under the condition of a wavelength to particle diameter ratio of 1.8. In each subfigure, the left, middle, and right columns refer to the gel–particle reference (Case 1), perfect bonding hard particles (Case 2), and insufficient bonding hard particles (Case 3), respectively. (**B1**–**B3**) Frequency domain monostatic waveform under the condition of a wavelength to particle diameter ratio of 1.3. (**C1**–**C3**) Frequency domain monostatic waveform under the condition of a wavelength to particle diameter ratio of 0.8. (**D1**–**D3**) Frequency domain monostatic waveform under the condition of a wavelength to particle diameter ratio of 0.3.

In Figure 6, the time domain first roundtrip and second roundtrip pulses from Figure 5 are converted into frequency spectrums. The subfigures in Figure 6 in rows correspond to subfigures A to D in Figure 5. With reference to in Figure 6(A1,B1,C1,D1), the middle column and right column refers to the perfect bonding condition and insufficient bonding condition, respectively. In Figure 6, from row A to row D, with the operating wavelength decreasing, the attenuation performance of the introduction of hard particles increases along the increase in pulse center frequencies. From Figure 6(A2,B2), the frequency spectrums showed slight interferences but still roughly followed Gaussian-like distributions, which proved that there were fewer contributions from the strong scattering or internal reflections when the wavelength was longer for the hard particles that perfectly bonded with the surrounding gel. When the bonding between hard particles and the surrounding gel became worse, more significant scattering and backscattering-induced interreference could be observed from the spectrums (Figure 6(A3,B3)). With respect to the hard particle size, shorter wavelength pulses propagated with much stronger interferences from the internal reflections, which made the frequency spectrums highly distinguishable with original Gaussian-like distributions (Figure 6(C2,D2)). In particular, such interferences presented as only destructive interferences. Potential constructive interferences are due to significant superposition between internal reflections and major roundtrip pulses, which makes conventional attenuation estimation invalid. Nevertheless, compared the previous experimental data on the micro-Graphite Agar and nano-Al_2_O_3_ Agar samples with the numerical simulation results, the difference between the collected acoustic wave propagation behaviors in the experiments was comparable to the difference between Figure 5C Case 3 and Figure 5B Case 2 and Figure 6(B1) and Figure 6(C2) in terms of both time domain and frequency domain behaviors.

## 4. Discussion

Acoustic properties of tissue phantoms are important to represent the acoustic wave propagation behaviors in real tissues. However, real tissues can be mechanically very different based on the tissue categories, health conditions, and even IBM values. Hence, acoustic property modifications are necessary in tissue phantom fabrications to mimic ultrasound images or ultrasonic detection practices. As known, acoustic attenuation refers to the acoustic energy loss during wave propagation, which is even more complex in real tissues due to the potential existence of skin, fat, fibrosis, bones, interfaces between different tissues, and other factors. The absorption, internal reflection, scattering, dispersion and divergence of the source wave can significantly lower the receiving echo. However, such differences in energy loss mechanisms have not been well distinguished in many tissue phantom studies [30,38]. As reported, adding liquids [21], air bubbles [39], or small solid particles [28,29] can increase the acoustic energy loss, or so-called acoustic attenuation, but the energy loss mechanisms can be highly different. Grouping the energy loss mechanisms in different tissue phantoms can be beneficial for future tissue phantom selection for specific ultrasound detection applications and used as additional characteristics besides elastic modulus and speed of sound values. However, such acoustic energy loss mechanisms are slightly challenging to observe in practical experimental approaches, and numerical simulations are more suitable for the detailed visualization and analysis of wave propagation behaviors.

Numerical simulations under controlled conditions were used to compare the effect of different particle sizes and particle bonding conditions on wave physical behavior. But agreements between the experimental observations and the numerical simulations only show facts such as the Graphite particles having larger sizes and worse bonding compared to the Alumina particles in an overall view. In the fabricated samples, some small-size Graphite particles or large-size Alumina particles might have also existed, but they were not the major populations.

As reported, adding oil or milk in the recipes of tissue phantom hydrogels increases the acoustic attenuation coefficients [21]; however, in addition to other studies, this study pointed out that the increase in total attenuation was not only caused by energy absorption but also caused by scattering and dispersion effects. Different to solid particles, the additional liquid in hydrogel recipes can be either dissolved or breakdown into small droplets (liquid cavities). Mixing well can decrease the additional acoustic attenuation caused by scattering and dispersion effects [30]. By adding solid phase small particles, mixing well can separate the accumulated clusters but it cannot break down typical-sized particles in general. Hence, the size of the additional solid particles, or in other words, the ratio between the operational wavelength and the typical particle sizes, plays a critical role in changing the contribution of acoustic energy loss mechanisms to the total acoustic attenuation.

By gaining a deeper understanding of the detailed energy loss mechanisms in the gels with hard particles, potential benefits could be obtained in ultrasound imaging or tissue phantom design. With scattering-dominant gel recipes, fewer dispersion effects can help researchers easily develop temporal-dependent gain functions to reduce energy loss in deep tissue imaging applications [40,41]. With dispersion-dominant gel recipes, frequency-dependent and spatially dependent dispersion can be evaluated on each specific gel recipe to optimize the suitable imaging frequency and suitable imaging depth [21,42]. For tissue phantom designs, in potential future studies, understanding the contributions of scattering and dispersion in tissue phantoms could help mimic tissue phantoms with different real tissues including soft tissues, hard tissues, fat-concentrated tissue, or tissues with fibrosis.

As some studies have suggested [31,32], the size of solid particles in tissue phantom gels has significant effects on the acoustic attenuation coefficient or absorption coefficient. Although the ratio between the operational wavelength and typical particle sizes has been barely mentioned, the important frequency dependence has been well determined by experimental and theoretical approaches. In addition, the selected acoustic operational wavelength values are generally much larger than the average particle sizes in the mentioned literature. High-frequency ultrasonic acoustic properties are not usually the focus. In this study, the conducted numerical simulations offer clear proof and an analysis of the literature observations with controlled-condition configurations, and they have the potential to help future designs of new tissue phantoms, soft resonators [43], ultrasound absorbers [30], and even soft sensors [44].

## 5. Conclusions

Additives are commonly applied to tissue phantom to increase acoustic attenuation for potential ultrasonic imaging and detection applications. However, detailed attenuation and acoustic energy loss mechanisms in different gel recipes have barely been discussed. In previous studies, the significance of dispersion-induced out-of-phase interference has been discussed in detail in tissue phantoms with 10 wt.% liquid additives, which strongly increase the overall attenuation effect without considering energy absorption and scattering effects. In this study, energy loss mechanisms were further estimated and discussed in detail in gels with 10 wt.% solid additives. In specific, nano-Alumina and micro-Graphite were found to provide highly distinguishable temporal waveforms and frequency spectrum distribution in the experimental time-of-flight measurements. To observe their contributions on the particle-to-wavelength ratios and particle–gel bonding conditions, controlled-condition numerical simulations were conducted, from a >1 particle-to-wavelength ratio to a <1 particle-to-wavelength ratio, with both a perfect bonding condition and insufficient bonding condition. Under the <1 particle-to-wavelength ratio condition, the gel presents stronger dispersion-induced energy loss than the gel with the >1 particle-to-wavelength ratio condition. On the contrary, the gel with the >1 particle-to-wavelength ratio condition shows more significant scattering-induced destructive interferences compared to the gel with the <1 particle-to-wavelength ratio condition. Under the insufficient bonding condition regarding the gel containing >1 particle-to-wavelength ratio particles and the good bonding condition regarding the gel containing <1 particle-to-wavelength ratio particles, the corresponding scattering effects and dispersion effects contributed to the overall energy loss more, respectively, which presents an agreement with the experimental measurements of the Agar gels with micro-Graphite and nano-Alumina particles, respectively.

## Data Availability

Data available from the requests to the corresponding author.
